# Bicloride of Mercury Baths in the Treatment of Variola

**Published:** 1909-08

**Authors:** R. H. L. Bibb

**Affiliations:** Saltillo, Mexico


### TEXAS MEDICAL JOURNAL.

Established July, 1885.

F. E. DANIEL, M. D., - - - - - Editor, Publisher and Proprietor

PUBLISHED MONTHLY.—SUBSCRIPTION $1.00 A YEAR.

VOL. XXV. AUSTIN, AUGUST, 1909. No. 2.

The publisher is not responsible for the views of contributors.

### Original Articles.

For Texas Medical Journal.

### Bicloride of Mercury Baths in the Treatment of Variola.

BY R. H. L. BIBB, M. D., SALTILLO, MEXICO.

Although epidemics of variola occur less frequently than in former times, and seem to be decreasing yearly, the ravages of the disease, and its loathsomeness, is such as to clothe any method designed to ameliorate its victims with sufficient importance to justify repetition on occasions such as is the present one. This shall constitute my excuse, if excuse is needed, for reproducing, in part, an article on this subject published by myself, in the TEXAS MEDICAL JOURNAL November, 1899.

Some twenty years ago my distinguished friend, the late Dr. T. C. Osborn of Cleburne, Texas, wrote me, in laudatory terms, of the potency of bichloride of mercury baths in the treatment of smallpox, which he had employed for many years, and for which he claimed marvelous results, urging me to let no opportunity pass for according its benefits to such of my patients as might fall victims to variola.

Dr. Osborn assured me that by prompt, timely and frequently sponging a variolous patient from head to foot with a bath as hot as the patient could bear it, of solution of bichloride of mercury in distilled water, one to one thousand and stronger, that the disease could be arrested before reaching the suppurative stage; that the intolerable itching and the insufferable odor so prominent in smallpox, and suppuration, with its many dangers, could be prevented; and that desquamation, with its scabs and its scales, being rendered aseptic, would no longer be a menace to homes and communities.

For a long time, much to my regret now, notwithstanding their high and authoritative source, I regarded these claims with extreme skepticism, for I believed that the doctor, although a physician of ripe and varied experience, and an observer of acute perception and much erudition, had attributed abortive cases of variola to the result of his treatment; and it was not until I took charge, as physician and surgeon in chief, of the American Hospital in the City of Mexico, in January, 1897, did I have the temerity to try Dr. Osborn's treatment, although I had many cases of smallpox in the meantime, and had never forgotten his eloquent appeals for its trial, nor his earnest assurances of its efficacy.

A few days after the date last written, I offered a young Frenchman, aged 22 years, to the American Hospital, with one of the most typical cases, and at the same time, one of the most violent, unmanageable and unpromising cases of smallpox that ever came under my observation—so violent, unmanageable and widely delirious was the patient that I was forced to order him the constant attention of two special male nurses, who, in order to control him, were obliged to strap him, hands and feet, to the bed. The disease was in the papulo-vesicular stage, the eruption literally covering the entire body, including the eyes, mouth, throat, nose and ears. He could swallow nothing. His temperature was high, his pulse was rapid and feeble, and his general condition indicative of little hope for recovery.

Believing the young man doomed—that no treatment would save him, I determined to give Dr. Osborn's treatment the benefit of the patient; should it fail to be of service to him, I was confident would be the result. Accordingly, he was ordered to be literally sponged, over the entire body, with a warm solution of bichloride of mercury, one to five hundred, in distilled water, every four hours; two centigrams of morphia, hypodermically, p. r. n.; his eyes, nose, mouth, ears and throat irrigated with a warm saturated solution of boric acid, in water, also a nutritive clyster of 200 grams of peptonized milk and egg, with 30 grams of brandy and 15 drops of laudanum every six hours.

At my regular visit, forty-eight hours after the treatment outlined above had been instituted, and which had been most faithfully and scrupulously administered, the nurse in charge, an old experienced smallpox nurse, informed me that whilst the patient was free from fever, had slept and had been quiet and rational from the night before, it not being necessary to restrain him longer, he thought he was worse in that the vesicles were shriveling up and turning black, as if it was a case of "variola-hemorrhagica," as he had never seen this phenomenon in any other form of the disease, although he had nursed hundreds of cases of smallpox, both in natives and in foreigners.

From a careful examination of the young man's general condition, the great improvement, as compared with his condition, on the previous evening, the complete absence of fever, of nervous symptoms and of hemorrhage from the kidneys, nose and other mucous membranes, I could easily, and did exclude "variola-hemorrhagica." This left me to decide whether the disease was aborting, as it sometimes does, or whether it was being favorably influenced by the treatment. I adopted the latter hypothesis, and as subsequent events and a more enlarged experience demonstrated, chose correctly, for my patient recovered rapidly, without smell, without pustulation, without secondary fever and other secondary complications, and, excepting a few pits about the forehead, nose and mouth, where the vesicles were ruptured and repeatedly irritated while cleaning these parts, without pitting or other disfigurement.

Thus, in brief outline, is the treatment and results in forty cases of smallpox treated at the American Hospital whilst I was in charge of that institution, and some sixty-nine additional cases treated outside during that time and up to the present writing.

In none of the 109 cases thus treated (I refer to the treatment wherein the bichloride of mercury baths, as detailed above, constituted the essential treatment addressed to the disease—of course, subsidiary treatment was used to combat individual symptoms in individual cases, such as diarrhoea, etc.), where treatment antedated pustulation, i. e., when it began before vesiculation, was there troublesome itching, pustulation, foul odors, pitting or secondary consequences of any character whatever—all, heretofore, much dreaded accompaniments of smallpox; and, in but one case, a female who entered the hospital with pustulation, and who was treated with the bichloride in tablet form (blue tablets)—I had always used red tablets or the bichloride salt—were there abscesses. I would call the attention of the profession, most especially to the fact that my experience with the bichloride in the form of blue tablets, is that it is absolutely inert. From what cause I do not know. Another precaution—be sure that the bichloride is not dissolved in a metallic vessel, as it will be precipitated.

Of the 109 cases treated as stated, eight have died, one a young man of alcoholic and syphilitic antecedents, 35 years of age, died of haematuria in the papillary stage of the disease twenty-four hours after admission into the hospital. Another, an old hospital nurse, 73 years old, the subject of Bright's disease, died of heart failure, also in the papillary stage of his illness, a few hours after the first sponging. I will state that it is my conviction that this patient died simply from the knowledge that he had smallpox. He died of heart failure. Of the others, all private patients, three died from pneumonia, one from diarrhoea and two from heart failure—one being a small child, aged 3, the other, aged 50 years, with a dilated heart; this, however, was a modified case.

Children and adults, male and female, were treated alike, i. e., with a solution of the same concentration, one of bichloride to five hundred of distilled water, and all with like happy results.

Stomatitis, which is not infrequent in variola, however treated, occurred in one case; and although the mercurial baths were continued, instead of increasing, as it most surely would have done if it had been caused by them, improved, pari passu, with improvement in the patient, which was a child, and nothing was used against it except the boric acid solution already mentioned.

I have never had an opportunity to determine, by inoculation experiments on calves, or otherwise, whether the scabs from patients thus treated, are sterile or not, as to the virus of variola and other pyogenic organism. One can reasonably imagine them to be, and should expect them to be, inasmuch as the treatment prevents suppuration, unless it can be shown that the presence of these organisms does not, of necessity, induce the pyogenic process.

The first change noted in the vesicles treated by the bichloride, as given above is that they become opalescent, as if their contained fluid had coagulated, and the surrounding zone of inflammation fades away, the vesicles speedily shrivel, their contents turn black, so that by the time that they are dry they resemble small tacks driven into the skin, and with desquamation, they fall out, as one patient put it, "like peas from the pod."

In my humble judgment, the following conclusions are warranted:

First. That the bichloride of mercury, applied as herein indicated and soon enough, is the rational treatment of variola.

Second. That its use will prevent itching, foul odors, pustulation, abscesses and pitting.

Third. That it will greatly lessen the mortality and suffering from one of the most loathsome diseases that afflicts the human race.

Fourth. That it should be expected to destroy the variolous virus in the vesicles, thus rendering the scabs and scales harmless, reducing thereby to a minimum the dangers of infection from a given case of smallpox.

Saltillo, Mexico, January 15, 1909.

